# Shiga toxin producing *Escherichia coli*-associated diarrhea and hemolytic uremic syndrome in young children in Romania

**DOI:** 10.1186/s13099-019-0327-4

**Published:** 2019-09-25

**Authors:** Oana Falup-Pecurariu, Raluca Ileana Lixandru, Emanuela Cojocaru, Katalin Csutak, Vlad Monescu, Khitam Muhsen, Cristian Falup-Pecurariu, Daniel Cohen

**Affiliations:** 1 Faculty of Medicine, Transilvania University, Brașov, Romania; 2 Department of Pediatrics, Children’s Clinic Hospital, Brașov, Romania; 3 Faculty of Mathematics and Informatics, Transilvania University, Brașov, Romania; 4 Department of Epidemiology and Preventive Medicine, School of Public Health, Sackler Faculty of Medicine, Tel Aviv University Ramat Aviv, Tel Aviv, Israel

**Keywords:** Gastrointestinal infection, Shiga toxin producing *E. coli*, Complication, Hemolytic uremic syndrome

## Abstract

**Background:**

Diarrheagenic *Escherichia coli* (*E. coli*) is an important cause of diarrheal diseases in both developing countries and industrialized countries. An outbreak of hemolytic uremic syndrome (HUS) in young children from southern Romania was reported in early 2016 and was attributed to Shiga toxin producing *E. coli* (STEC) O26 infection. The aim of this study was to determine the prevalence, demographic and clinical characteristics of STEC infections in children hospitalized with diarrhea in Brașov in the central region of Romania. We also described the occurrence of HUS among hospitalized children, close in time to the 2016 HUS outbreak in southern Romania.

**Methods:**

A prospective study was conducted between March and December 2016 among 722 children aged 1–30 months hospitalized with acute diarrhea. Stool samples obtained from patients with diarrhea were tested for the presence of Shiga toxin type 1 (STX1) and type 2 (STX2) by an immunochromatographic assay, and other enteropathogens. Demographic and clinical information on cases of HUS diagnosed in the same hospital was obtained from medical records.

**Results:**

Overall 46/722 (6.4%) children (mean age 10.3 months, 32.6% females) hospitalized with diarrhea tested positive for STX1 or STX2; of these 79% were positive for both STX1 and STX2, 16% for STX2 only, and 5% for STX1 only. Bloody diarrhea, vomiting and fever were documented in 32.6%, 52.1% and 50.0%, respectively of patients with STEC infection. Eleven confirmed HUS cases (mean age 20 months, five females) were identified between 2014 and 2016 with prodromal diarrhea reported in 10 of them. Three of the 11 HUS patients required hemodialysis.

**Conclusions:**

STEC prevalence among young children with diarrhea in Romania was high and the risk of HUS is emerging. The establishment of a systematic laboratory-based surveillance program including identification of the circulating STEC strains coupled with epidemiological investigation of HUS patients is warranted to determine the source and mode of transmission of STEC and prevent of STEC-associated diarrhea and HUS.

## Background

Diarrheagenic *Escherichia coli* (*E. coli*) strains are classified in six major groups [1, 2] based on the characterization of virulence genes and an important group is Enterohemorrhagic *E. coli* (EHEC). EHEC strains are unique in their ability to produce Shiga toxin type 1 (STX1) and type 2 (STX2) encoded by *stx1* and *stx2* genes, respectively, which cause non-bloody and bloody diarrhea as a result of a cytotoxic effect on the capillary endothelium [1–4]. Human virulent Shiga toxin producing *E. coli* (STEC) strains may often contain genes encoding to other virulence factors such as intimin (*eae*), and *E. coli* hemolysin (*ehxA*) [5].

STEC-associated diarrhea may have complications, including hemolytic uremic syndrome (HUS) that develop in about 5–10% of the patients [6]. Virulence gene profile of the bacterium and young age (i.e. children less than five years of age) are the major determinants of HUS [7].

The most common STEC strain is *E. coli* O157:H7. Non-O157 strains that account for 20–50% of STEC strains in the United States are estimated to cause 36,000 illnesses, 1000 hospitalizations and 30 deaths annually in the United States [8]. There are countries including Argentina, Australia, and Germany in which non-O157 STEC serogroups dominate over O157 serogroups [9]. Non-O157 serotypes appear to produce watery diarrhea more often than bloody diarrhea [10].

The STEC strains are the third cause of bacterial diarrhea after *Campylobacter* and *Salmonella* in several industrialized countries [11]. The epidemiology of STEC infection is not fully clear, although outbreaks caused by STEC have been reported [9]. Humans are normally infected with STEC through the fecal–oral route [12]. The major vehicles of STEC transmission are unpasteurized milk and juice along with uncooked beef, cattle’s meat and seeds or vegetables contaminated with fecal material containing STEC [13, 14].

There is major public health concern regarding STEC-associated diarrhea, given the potential of causing massive outbreaks and HUS, and even fatalities [15, 16]. For example, in 2011 a large outbreak caused by STEC of serotype O104:H involving over 4000 cases of bloody diarrhea, more than 800 cases of HUS and 50 deaths, affected various countries in Central Europe and had the epicenter in Germany [14]. The massive transmission of the epidemic agent was foodborne, vehicles were sprouts, which germinated from fenugreek seeds [17]. In January–February 2016, an outbreak of HUS was reported in Romania, involving 15 children with HUS and three of them died. Most patients were less than two years of age from southern Romania. The etiology was retrospectively confirmed through serological testing, showing that six patients had STEC O26 infection [18]. All HUS patients had initially presented with diarrhea and some with bloody diarrhea [18]. Evidence on the prevalence of STEC among children with sporadic diarrhea, outside the context of outbreak investigation, remains limited. Accordingly, the aim of the present study was to determine the prevalence of STX1 and STX2 infections in children hospitalized with diarrhea in Brașov, in the central part of Romania, close in time to the 2016-HUS epidemic in southern Romania [18]. We also examined the demographic and clinical characteristics of STEC-associated diarrhea and described the occurrence of HUS hospitalizations during 2014–2016.

## Results

Overall, stool samples were obtained from 722 children hospitalized with acute diarrhea between March 1st 2016 and December 31st, 2016; of these 172/722 (23.8%) tested positive for rotavirus, 19/722 (2.6%) were positive for adenovirus (of these four children had mixed infections with adenovirus and rotavirus); one child (0.1%) was positive for *Shigella* spp. and one (0.1%) for *Salmonella* spp. All samples were negative for *E. coli* O 157 when cultured on Sorbitol-MacConkey-plates. STEC was detected among 46/722 (6.4%) children who tested positive for STX1 or STX2; of these, 79% were positive for both STX1 and STX2, 16% for STX2 only, and 5% for STX1 only.

Demographic, clinical and microbiological characteristics of 46 patients with STEC infection are given in Table 1. Bloody diarrhea was documented in 15/46 (32.6%), vomiting in 24/46 (52.1%) and fever (≥ 38 °C) in 23/46 (50.0%). Thirteen (28.2%) and seven (15.2%) of the 46 children with STEC had mixed infections with rotavirus and adenovirus, respectively and the rest had single STEC infection. Vomiting was more common in children with mixed STEC plus rotavirus infections 10/13 (77%) versus those with STEC infection who did not have rotavirus 13/32 (41.0%), (p = 0.047 by Fisher’s exact test] and adenovirus plus STEC [6/7 (85.7%) versus those who had STEC without adenovirus 16/37 (43.2%), (p = 0.09)]. No significant differences were found for any of the other parameters examined.

**Table 1:** Demographic, clinical and microbiological characteristics of 46 children aged 1–30 months hospitalized with acute diarrhea and STEC infection

Characteristics	Number (%)
Sex	
Males	31 (67.4%)
Females	15 (32.6%)
Age (months)	
Mean (standard deviation)	10.3 (6.5)
1–5	13 (28.3%)
6–11	15 (32.6%)
12–30	18 (39.1%)
Ethnicity	
Caucasian	29 (63.0%)
Roma	17 (37.0%)
Gestational age at birth	
Term delivery	40 (87.0%)
Low grade premature	6 (13.0%)
Birth weight (kg)	
2.2–2.4	6 (13.0%)
2.5–4.2	40 (87.0%)
Clinical manifestation	
Diarrhea	46 (100.0%)
Bloody diarrhea	15 (32.6%)
Vomiting	24 (52.2%)
Fever ≥ 38 °C	23 (50.0%)
White blood cells (cells/mcL)	
< 10,000	28 (60.9%)
≥ 10,000	18 (39.1%)
Neutrophils (cells/mcL)	
1400–4600	23 (50.0%)
4601–13,000 cells/mcL	23 (50.0%)
Hemoglobin, gr/dL	
< 11	17 (37.0%)
≥ 11	29 (63.0%)
C reactive protein	
< 1	44 (95.7%)
≥1	2 (4.3%)
Duration of hospitalization (days)	
Minimum–maximum	1–14
Median (interquartile range)	5 (2)
Mixed infection	
With rotavirus	13 (28.2%)
With adenovirus	7 (15.2%)
With *Salmonella*	1 (2.1%)

One of the 46 children (female aged 10 months) with STEC-diarrhea (positive for STX2) developed HUS. She was admitted to the hospital with bloody diarrhea and seizures, intra-rectal temperature of 37.2 °C. White blood cells count at admission was 20,500 cells/mcL (normal range 4000–10,000 cells/mcL), hemoglobin of 8.9 g/dL (normal range 12–14 g/dL), hematocrit of 22.2% (normal range 34–35%), thrombocytopenia (45,000 cells/mcL) (normal range 150,000–400,000 cells/mcL), creatinine of 3.06 mg/dL (normal value < 1.2 mg/dL), urea of 135 mg/dL (normal value 20–40 mg/dL). The patient was transferred for hemodialysis.

## Cases of HUS hospitalized between 2014 and 2016

**Fig. 1:**
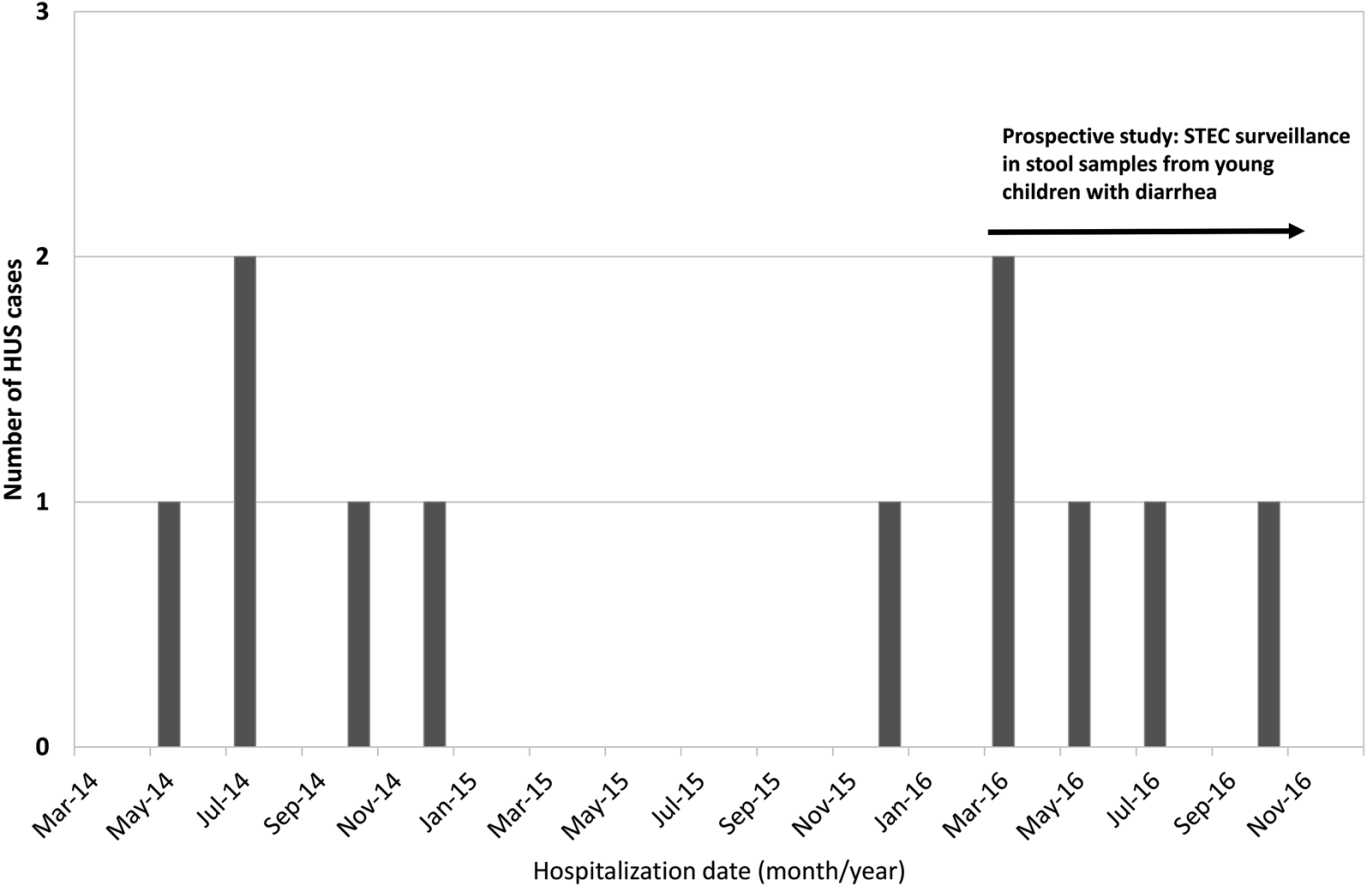
Number of hospitalized patients with hemolytic uremic syndrome by month, 2014 and 2016 in Brașov, Romania

Screening of the hospital medical records led to the identification of 10 cases of HUS (Fig. 1). The age of the 11 confirmed HUS cases (including the case with documented STEC-diarrhea in 2016) ranged from 5 to 30 months (median age: 10 months). Six cases were females. Prodromal diarrhea was reported for 10 cases, and four had bloody diarrhea. Vomiting and fever (≥ 38 °C) were reported for nine and four cases, respectively. All cases had hematuria and proteinuria, and five cases had oliguria (Table 2). Eight HUS patients had white blood cells count of 15,000/mcL or higher. All had thrombocytopenia (mean platelet count 73,000 cells/mcL (range 37,000 to 138,000 cells/mcL). The mean hemoglobin level at admission was 9.2 g/dL (standard deviation 1.8). Ten patients had the hematocrit lower than 30.0% (mean in11 HUS cases: 24.7%, range 17.2% to 30.2%) and all had serum creatinine levels above the normal values of ages 0–2 years (range 0.86–3.64 mg/dL). Five HUS patients showed increased lethargy, eight had marked pallor and one had seizures (Table 2). Three of the 11 patients were transferred for hemodialysis.

**Table 2:** Demographic, clinical and laboratory characteristics of children aged 5–30 months hospitalized with hemolytic uremic syndrome, Brașov, Romania, 2014–2016

Total number of patients	11
Age	Mean: 20 months
	Median: 10 months
	Range: 5–30 months
Female/male ratio	6/5
Prodromal diarrhea	10
Bloody diarrhea	4
Vomiting	9
Fever ≥ 38 °C	4
Hematuria	11
Proteinuria	11
Oliguria	5
White blood cells count ≥ 15,000 cells/mcL	8
Trombocytopenia (mean 73,000 cells/mcL)	11
Hematocrit < 30%	10
Serum creatinine levels ≥ 3.64 mg/dL	11

Data presented are absolute number unless mentioned otherwise

## Discussion

We determined the prevalence of STX1 and STX2 infections in children hospitalized with diarrhea in Brașov in the months following the HUS cluster reported in southern Romania and analyzed the clinical and demographic characteristics of STX1 and STX2 infections. We also identified cases of HUS among children admitted to Brașov hospital close in time to 2016-HUS outbreak in southern Romania caused by STEC O26 as documented by serology [18].

We found a high prevalence of STX1 and STX2 infection (6.4%) in children aged 1–30 months hospitalized for acute diarrhea at the Children’s Clinic Hospital, Brașov between March 1st and December 31st, 2016. One of these patients developed HUS. Ten additional hospitalized patients with HUS, were identified between 2014 and 2016. This included four cases of HUS occurring in 2016 in the months after the epidemic peak of HUS reported in February 2016 [19] and after the recall on a food item (a particular type of cheese produced by a milk processing establishment in Romania) suspected as being the common vehicle of the epidemic agent [20].

Altogether, our findings indicate that there is continuous exposure to STEC strains in Romania possibly from multiple sources in the food chain in the region, leading to STEC-diarrhea and occurrence of sentinel sequelae such as HUS in very young children. Our study does not provide evidence on the STEC serotype secreting STX1 and/or STX2 that caused the STEC-diarrhea in the hospitalized children. Since *E. coli* O157 was not detected in any of the stool samples cultured on Mac Conkey-Sorbitol, non-*E. coli* O157 verotoxigenic strains likely were the cause of STEC-diarrhea and HUS in patients admitted to our center.

The onset of the major epidemic of HUS in Romania linked presumably to STEC O26 raised important questions related to the extent of circulation of this and other STEC strains in Romania and Europe and the potential occurrence of additional cases of HUS linked to these STEC strains.

In Europe and in the United States, the role of non-O157 STEC strains (e.g., O26:H11/H^−^, O91:H21/H^−^, O103:H2, O111:H^−^, O113:H21, O121:H19, O128:H2/H^−^, and O145:H28/H^−^) as causes of HUS, bloody diarrhea, and other gastrointestinal illnesses is being increasingly recognized partly due to improved diagnostics of non-O157 sero-pathotypes [12, 21]. This trend culminated with the severe STEC O104 outbreak that took place in Germany and France in 2011 during which almost 4000 cases and more than 50 deaths were reported [14]. Reporting of STEC O26 infections has been steadily increasing in Europe since 2007, and in 2013 an additional outbreak of HUS in young children in Italy was retrospectively linked to this STEC serotype [18, 22, 23]. However, to-date evidence regarding STEC diarrheal disease in children in Romania remains limited.

The methodology of direct STX1 and STX2 identification in stool samples by antigen or respective virulence gene detection have increased the sensitivity of STEC-associated diarrhea diagnosis. The prevalence of 6.4% of STX1 and STX2 in our study among children with diarrhea aged 1–30 months examined between March and December 2016 is higher than that reported by other studies [11, 24, 25]. A prevalence of 2.4% was reported by Klein et al. [24] in a pediatric hospital in Seattle in the United States in 1998–2001. A study from Canada showed 2.7% prevalence in a 10-week survey conducted in 1990 on stool samples of patients (all ages) with diarrhea submitted for culture for diarrheal pathogens [25]. Over more than six years (from October 1992 to August 1999), 5054 samples from inpatients and outpatients of all ages that were submitted to the clinical microbiology laboratory for routine pathogen identification were screened for STEC in Lugo, Spain. The overall identification rate of verotoxins 1 and 2 in this period and encompassing various detection methods was 2.5% [26]. Buvens et al. [11] examined in Belgium the presence of STEC in diarrheal disease patients of all ages using multiplex PCR on *E. coli* colony sweeps, showing a 5.1% prevalence in patients with bloody diarrhea, and reported significantly higher prevalence of STEC in children compared with adults. A recent study conducted in India using PCR to detect STX1 and STX2 in lysates of *E. coli* from diarrheal patients of all ages reported a 15% prevalence for STX1 and STX2 [27], higher than that found in our study.

We assume that the high prevalence of STEC-diarrhea in our study is the result of high exposure to STEC strains and the young age of the diarrhea patients who had significant symptoms and signs of disease including one patient who developed HUS. One-third of the cases of STEC-diarrhea in our study (median age = 10.3 months) had bloody diarrhea at admission to hospital, about 50% vomited and had fever higher ≥ 38 °C. Similar findings were reported by other studies that characterized STEC infection in young children [26, 28, 29]. It has been shown that presence of STX2 was associated with a more severe disease compared to STX1 [30]. In our study, the combination of STX1 and STX2 was the most encountered followed by STX2 alone. This is one particularity compared to the published data.

Co-infections of STEC with *Campylobacter, Salmonella* and *Clostridium* were reported [1]. We found co-infections with rotavirus and adenovirus and with *Salmonella* in one case [1]. Interestingly, we found that vomiting on admission was in excess in subjects with co-infections of STEC with rotavirus. Since rotavirus has a very high pathogenicity especially in children under two years of age, we cannot rule out the possibility that in these patients STEC had a secondary pathogenic impact.

In our study, the mean age of STEC- diarrhea patients was 10 months, i.e., around the weaning period and thus the time, the young age and immunologically naïve children become increasingly exposed to foodborne transmitted pathogens including diarrheagenic *E. coli* [31].

In 10 of the 11 cases of HUS with onset of disease in 2014–2016, a prodromal diarrheal disease was documented. All fulfilled the most stringent definitions of HUS displaying the classic triad combining thrombocytopenia, hemolytic mechanical anemia, and acute kidney injury [6]. This may suggest an underestimation of the burden of STEC associated significant complications albeit with a milder manifestation.

Our study has some limitations. First, it is a study that covered only 9 months and it comprised only admitted patients. Secondly, the study did not include testing of *E. coli* isolates from stools positive for STX1 and STX2, to further identify non-*E. coli* O157 STEC serotypes secreting the verotoxins.

We evaluated the involvement of STEC in severe diarrhea leading to hospitalization of young children and onset of HUS in central Romania close in time with the 2016-HUS outbreak in children from southern Romania. This is to our knowledge the first report from Romania in which children aged less than 2.5 years hospitalized for diarrhea and other gastrointestinal complaints were systematically assessed for STEC etiology and HUS complication.

## Conclusions

The high prevalence of STX1 and STX2-associated diarrhea in hospitalized children with acute diarrha in 2016 and the identification of 11 cases of HUS with prodromal diarrhea between 2014 and 2016 (five in 2016) indicate significant exposure of infants and toddlers to STEC strains in the Brașov region. It also indicate that there is continuous risk of severe diarrhea with significant and sometimes irreversible complications such as HUS. A systematic laboratory-based surveillance program including identification of the circulating STEC strains and molecular characterization coupled with epidemiological investigation with analytical components on each HUS case should be implemented towards identification of the source and mode of transmission of STEC and prevention of STEC-associated diarrhea and HUS. The burden of cases of HUS is of much concern because of the severity of the diseases and the risk for permanent kidney failure and even death.

## Methods

### Study design and population

A prospective study was conducted between March 1st 2016 and December 31st 2016 to examine the prevalence of STEC in hospitalized children with acute diarrhea at the Children’s Clinic Hospital Brașov, Romania.

Brașov is located in the central part of Romania and has a population of 260,000 inhabitants. The Children’s Clinic Hospital Brașov is the biggest pediatric medical center in the county with 169 beds. During 2016 there were more than 33,000 presentations at the Emergency Department. Of 9970 hospitalized children, 1569 (15.7%) patients had diarrheal disease (over four unformed/watery/loose stools per day). Overall 1337 children with acute diarrhea were hospitalized between March 1st and December 31st  2016, and stool samples were obtained from 722 (54.0%).

In addition, to identify HUS-related hospitalizations among children in Brașov close in time to the 2016-reported HUS outbreak in southern Romania [18], the hospitalization records in the period 2014–2016 were retrospectively scrutinized. HUS was defined following the criteria of the United States Centers for Disease Control and Prevention [32], as micro-angiopathic nonimmune hemolytic anemia associated with acute renal failure (acute onset), and thrombocytopenia (thrombocytes below 80,000 cells/mcL). Renal injury was evidenced by either proteinuria or hematuria or elevated creatinine levels (greater ≥ 1.0 mg/dL in a child less than 13 years).

### Data collection

Information was obtained from laboratory records (see laboratory methods) on the identification of enteric pathogens in stool samples collected from children hospitalized for diarrheal disease. For patients with STX1 or STX2 positive stool samples in the prospective study and those with HUS in the retrospective study detailed demographic, clinical and laboratory data were collected from medical records, including on age, sex, symptoms (e.g., diarrhea, vomiting, fever, bloody stool), complete blood count, creatinine levels, and urine analysis.

### Laboratory methods

Stool samples were cultured for *Salmonella* spp. and *Shigella* spp. *Campylobacter* ssp. by standard methods and additionally plated on sorbitol Mac Conkey medium without tellurite or cefixime for detection of non-sorbitol-fermenting *E. coli* strains. Presence of rotavirus and adenovirus antigens in stool samples was determined by commercial immunochromatography kits. We used the CerTest EHEC STX1 + STX2 (CerTest Biotec, Bioscience SL, Zaragoza, Spain) kit based on the principle of a qualitative immunochromatographic assay employing monoclonal antibodies for direct detection of STX1 and STX2 produced by STEC in stool samples. The manufacturer reported both sensitivity and specificity of ~ 99% when the CerTest *E. coli* O157 + STX1 + STX2 immunochromatographic test was compared with the established and commercially available kit Shiga Toxin Quik Chek, TechLab^®^ for STX1 and STX2. The concordance between the results obtained by the two kits was excellent as documented by the very high Cohen’s kappa coefficient. Shiga Toxin Quik Chek, TechLab^®^ kit is also based on monoclonal antibodies to STX1 and STX2 that was validated against the classical Vero cell assay.

### Data analysis

Descriptive statistics were employed using frequencies and percentages for categorical variables and mean and median values for continuous variables. Differences between patients having mixed STEC infection with rotavirus or adenovirus versus those with STEC but lacking rotavirus/adenovirus in clinical characteristics were examined using Fisher-Exact test. Data were analysed using IBM SPSS version 25 (Armonk, New York, USA).

## Data Availability

The datasets during and/or analysed during the current study available from the corresponding author on reasonable request.

## Abbreviations

*E. coli*: *Escherichia coli*
EHEC: Enterohemorrhagic *Escherichia coli*
HUS: hemolytic uremic syndrome
STEC: Shiga toxin producing *Escherichia coli*
STX1: Shiga toxin type 1
STX2: Shiga toxin type 2
